# Detailed Topology Mapping Reveals Substantial Exposure of the “Cytoplasmic” C-Terminal Tail (CTT) Sequences in HIV-1 Env Proteins at the Cell Surface

**DOI:** 10.1371/journal.pone.0065220

**Published:** 2013-05-27

**Authors:** Jonathan D. Steckbeck, Chengqun Sun, Timothy J. Sturgeon, Ronald C. Montelaro

**Affiliations:** 1 Center for Vaccine Research, University of Pittsburgh, Pittsburgh, Pennsylvania, United States of America; 2 Department of Microbiology and Molecular Genetics, University of Pittsburgh School of Medicine, Pittsburgh, Pennsylvania, United States of America; University of Massachusetts Medical Center, United States of America

## Abstract

Substantial controversy surrounds the membrane topology of the HIV-1 gp41 C-terminal tail (CTT). While few studies have been designed to directly address the topology of the CTT, results from envelope (Env) protein trafficking studies suggest that the CTT sequence is cytoplasmically localized, as interactions with intracellular binding partners are required for proper Env targeting. However, previous studies from our lab demonstrate the exposure of a short CTT sequence, the Kennedy epitope, at the plasma membrane of intact Env-expressing cells, the exposure of which is not observed on viral particles. To address the topology of the entire CTT sequence, we serially replaced CTT sequences with a VSV-G epitope tag sequence and examined reactivity of cell- and virion-surface Env to an anti-VSV-G monoclonal antibody. Our results demonstrate that the majority of the CTT sequence is accessible to antibody binding on the surface of Env expressing cells, and that the CTT-exposed Env constitutes 20–50% of the cell-surface Env. Cell surface CTT exposure was also apparent in virus-infected cells. Passive transfer of Env through cell culture media to Env negative (non-transfected) cells was not responsible for the apparent cell surface CTT exposure. In contrast to the cell surface results, CTT-exposed Env was not detected on infectious pseudoviral particles containing VSV-G-substituted Env. Finally, a monoclonal antibody directed to the Kennedy epitope neutralized virus in a temperature-dependent manner in a post-attachment neutralization assay. Collectively, these results suggest that the membrane topology of the HIV gp41 CTT is more complex than the widely accepted intracytoplasmic model.

## Introduction

The envelope (Env) protein of HIV, which is the major virally-encoded protein present on the surface of the virion, is the primary target of the humoral immune response [1]. Env is composed of two subunits translated as a 160 kD polyprotein that is post-translationally cleaved to yield the highly glycosylated gp120 (or surface unit - SU) protein and the transmembrane (TM) protein gp41 [2]. In addition, gp41 is composed of three distinct domains: the ectodomain, which drives the membrane fusion process; the membrane spanning domain (MSD) that is thought to anchor Env in the membrane; and the C-terminal tail (CTT). gp120 functions to mediate binding to the primary receptor, CD4, and the coreceptor, primarily CXCR4 or CCR5, while gp41 mediates fusion of the viral and cellular membranes, resulting in infection [2].

The gp120 protein and the gp41 ectodomain have been extensively studied, both structurally and functionally, as they appear to be the important targets of the antibody response in infected individuals [1]. Likewise, the gp41 MSD is the focus of intensive study to determine the exact sequences involved in spanning the cellular and viral lipid bilayers [3]–[8]. The CTT, on the other hand, has largely been studied at a functional level, and has been demonstrated to play a role in viral Env incorporation [9]–[12], virion maturation [13]–[16], cellular Env trafficking [17], [18], and more recently, as a modulator of Env gp120 conformation on both the cell and virion surfaces [19], [20]. However, relatively little is known about the structure of the CTT aside from characterizations of peptide analogs of CTT subdomains, known as the lentivirus lytic peptides (LLPs), that have been demonstrated to be predominantly helical in membrane and membrane-mimetic environments [21]–[23].

The topology of the CTT has been largely ignored as a topic of research in the otherwise extensively studied field of Env structure. The prevailing model is that Env gp41 exists exclusively as a type 1 membrane protein, with an extracellular (or extravirion) N-terminal domain (the ectodomain), a single (helical) transmembrane domain, and a cytoplasmically-localized approximately 150 amino acid long C-terminal domain [3]. Early support for this model was provided by studies examining sequence and structural comparisons with other retroviral Env proteins, particularly the oncogenic retroviruses that have a single transmembrane domain followed by a short cytoplasmic tail [24]. The cumulative results from the majority of studies of CTT function support its localization in the cellular cytoplasm, consistent with the traditional model [9]–[13], [17], [18], [25]. More recently, an alternative topology for the CTT has been proposed based on reactivity of viral particles [26]–[28] and Env-expressing cells with a monoclonal antibody (MAb) directed to the CTT [26]–[29]. Early data consistent with an alternative topology was published in the 1980s when Kennedy and colleagues discovered that serum from rabbits immunized with a peptide from the gp41 CTT could neutralize virus [30], and that antibodies reactive to that particular peptide were found in HIV-infected humans [31], [32]. These results suggested exposure of the peptide epitope, known as the Kennedy epitope (KE), on the virion surface, as antibody cannot cross intact lipid membranes.

More recent studies have yielded interesting, if sometimes conflicting, results regarding CTT topology. Initial studies of CTT topology by Dimmock and colleagues suggested that the KE was exposed on both the cellular and viral surface [33], [34]. Later experiments, however, demonstrated that the KE appeared to be exposed on the cell, but not the virion [26], [27]. Our lab has recently published similar results demonstrating the exposure of the Kennedy epitope on the surface of intact Env-expressing cells, but no apparent exposure on the virion [29]. These cellular results are in contrast to a study using GFP-fused gp41 truncation mutants where gp41 CTT was only observed in a cytoplasmic orientation in Env-expressing cells [35]. More recently, Desrosiers and colleagues have observed apparent exposure of CTT sequences on the surface of Env-expressing cells [36]. However, their results suggested that apparent CTT exposure was due to “shedding” of Env from expressing cells that subsequently bound to the surface of non-expressing cells and not that the CTT was natively exposed [36]. Attempts to interpret and condense the results of these studies into a topological model for the CTT are hindered by the limitations of the differing techniques used by each group to determine CTT topology.

Complicating CTT topology studies even further are results suggesting that the CTT undergoes dynamic rearrangements during the fusion process. This was first observed using the post-attachment neutralization (PAN) assay and finding that an anti-KE MAb, SAR1, could neutralize HIV when preincubated with virus and cells at 25°C, but did not neutralize either at 37°C (in the PAN format) or in a traditional neutralization assay [26], [27]. A more recent study has also indicated that topological rearrangements of the CTT take place during the membrane fusion process. Using an antibody directed to LLP2, Chen and colleagues found that they could detect binding directed to the CTT (LLP2) if cell-cell fusion was carried out at 31.5°C, but not at 37°C [37]. These results are analogous to those obtained with SAR1 using the PAN assay in that CTT sequence exposure is only observed when the fusion process is delayed using a lower temperature, suggesting that the apparent exposure during fusion at physiological temperatures is likely to be highly transient. This model is consistent with data demonstrating that the LLP2 sequence is membrane associated both during and after membrane fusion [38]. Together, the historical published results present an uncertain picture of CTT membrane topology.

In an effort to provide a comprehensive experimental CTT topological map, we have extended previously published experiments [29] using the VSV-G epitope tag insertion technique to determine the reactivity of the entirety of the CTT sequence, both on the surface of Env-expressing cells and on viral particles. The results of the current studies reveal surface exposure of the majority of the CTT in a portion of the Env expressed in intact transfected or infected cells. On the contrary, no apparent exposure of CTT sequences was observed on pseudoviral particles containing VSV-G-tagged Env. These results provide further evidence for a complex and heterogeneous CTT topology beyond the widely accepted intracytoplasmic model.

## Results

### The majority of the CTT sequence is exposed on the cell surface

**Figure 1:**
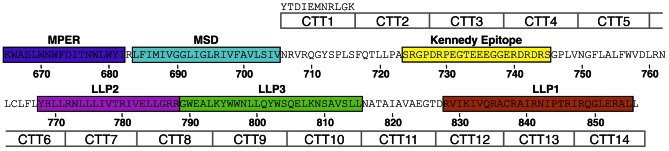
Replacement of HIV gp41 sequences with VSV-G sequences. The VSV-G epitope tag was used to replace gp41 CTT sequences serially across the length of the CTT as indicated, from CTT1 to CTT14. The VSV-G epitope tag sequence is listed above CTT1.

To map the topology of the CTT, a VSV-G epitope tag was used to serially replace the native sequences over the entirety of the CTT [29], [39]–[41] (Figure 1). Additionally, the HA epitope tag was inserted into the V3 region of gp120 to allow for detection of the relative levels of cell surface Env in cells transfected with the various VSV-G substituted Envs. The resulting constructs were then used in assays to determine reactivity with Env on the surface of intact live cells, or on the surface of pseudovirus particles (see below).

**Figure 2:**
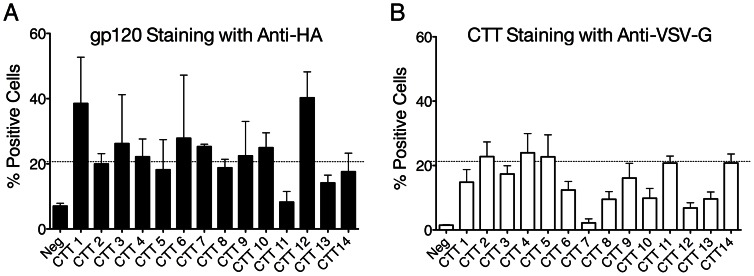
HIV Env staining of intact live cells. HEK293T/17 cells transfected with the indicated VSV-G containing Env were stained with (A) anti-HA, and (B) anti-VSV-G monoclonal antibodies. Only intact live cells were selected for analyses of Env exposure to reference monoclonal antibodies. (A) Total cell surface Env expression was determined by staining cells with anti-HA, targeting HA epitope tag inserted into gp120. (B) CTT exposure was determined by staining cells with anti-VSV-G, targeting the VSV-G epitope tag in the CTT sequence as demonstrated in Figure 1. The line at 20% staining is for visual reference only.

VSV-G-labeled Env constructs were transfected into HEK293T/17 cells and analyzed for epitope tag reactivity to either anti-HA or anti-VSV-G MAbs as described previously [29]. Initially, cells were dual stained with anti-HA and anti-VSV-G, however, no HA (gp120) reactivity was observed in spite of positive VSV-G staining. It was determined by separate staining for HA and VSV-G reactivity in aliquots from the same transfection that the anti-VSV-G (CTT) MAb was apparently inhibiting the binding of the anti-HA MAb. Therefore, to determine overall levels of Env expression on the cell surface, transfected cells were first stained for reactivity with anti-HA MAb (Figure 2A). In general, staining of all CTT constructs resulted in between 20% to 40% of cells labeling positively for gp120 on the surface of intact non-permeablized cells, with the exceptions of CTT11 and CTT13. To determine VSV-G, and thus CTT, exposure on the cell surface, separate aliquots of the respective transfected cells stained with anti-HA (gp120) were stained in parallel with anti-VSV-G (Figure 2B). In general, a similar overall level of staining was observed with the anti-VSV-G MAb, with the majority of CTT constructs showing approximately 20% positive labeling, with the exception of CTT 6, 7, 8, 12, and 13. Results with CTT3 and CTT7 are consistent with results obtained previously with these constructs [29]. These results suggest that the majority of the CTT sequence of gp41 is exposed and reactive to antibody binding on the surface of live intact, non-permeabilized Env-expressing cells.

**Figure 3:**
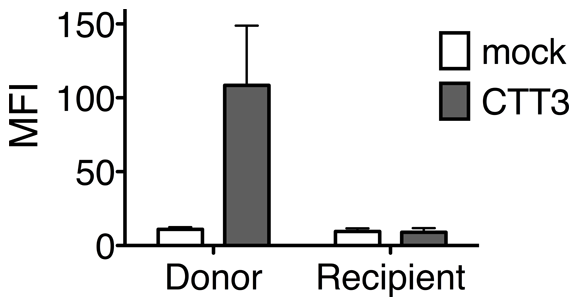
CTT reactivity does not transfer from Env-expressing cells to Env-naïve cells. Transfected cells (mock and CTT3) were stained to determine cell surface anti-VSV-G reactivity (labeled Donor). Clarified supernatants from the Donor cells were transferred to the Recipient cells as described [36], and the Recipient cells were stained to determine anti-VSV-G reactivity. Only Donor cells expressing CTT3 demonstrated staining with anti-VSV-G MAb. Cell surface CTT was not detected on the Recipient cells incubated with supernatant from CTT3 Donor cells.

To address the recent report that CTT-exposed Env is likely due to binding of Env shed from expressing cells to the surface of other cells [36], clarified supernatants from Env-expressing cells were transferred to untransfected cells, and the cells were then assayed for anti-CTT MAb binding. Cells transfected with CTT3 demonstrated binding of anti-VSV-G MAb (Figure 3, “Donor”). However, anti-VSV-G MAb binding was not detected to cells incubated with clarified supernatant from CTT3-transfected cells (Figure 3, “Recipient”). These results indicate that under the current experimental conditions, apparent CTT cell surface exposure cannot apparently be attributed to nonspecific shedding of Env from transfected cells to the surface of other cells.

### CTT-exposed Env constitutes a large proportion of cell-surface Env

**Figure 4:**
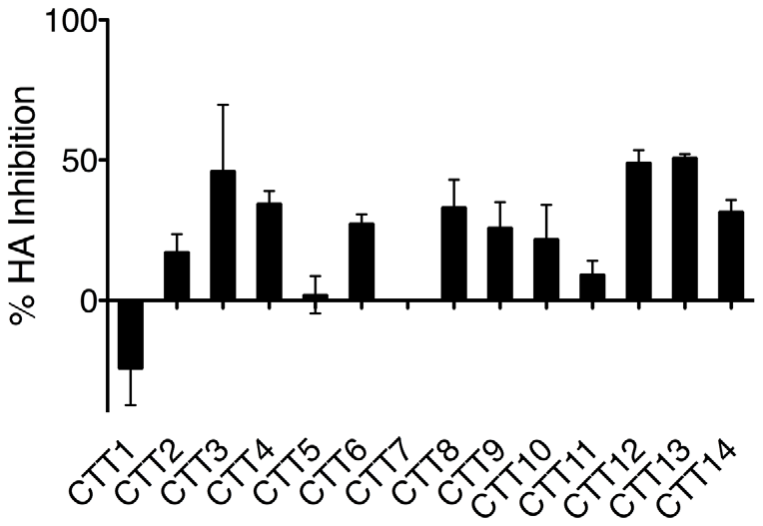
Inhibition of HA-specific antibody binding by VSV-G-specific antibody. Env-expressing cells were first reacted with unlabeled anti-VSV-G (CTT) antibody followed by staining with fluorescently-labeled anti-HA (gp120). The percent reduction in HA-labeled cells compared to HA-stained cells without VSV-G staining is presented as percent HA-inhibition.

While the above experiments demonstrated that a majority of the CTT sequence appears to be exposed on the surface of intact Env-expressing cells, they provide no information on the proportion of CTT-exposed Env relative to the total Env population. In order to determine the relative extent of CTT-exposed Env, we took advantage of the previous observation that anti-VSV-G (anti-CTT) antibody prevented anti-HA (anti-gp120) binding during attempts at dual staining for FACS analysis. The rationale for this experiment is that blocking of anti-gp120 antibody binding by anti-CTT antibody allowed a relative quantitation of CTT-exposed Env as a function of the total Env population. As total Env cell surface expression levels differed between each construct (Figure 2A), results were normalized to total HA binding in the absence of VSV-G antibody to allow a comparison between constructs. Results are presented as the percent HA binding inhibited by VSV-G antibody (Figure 4). The majority of constructs resulted in >20% inhibition of gp120 binding, with CTT3, CTT12, and CTT13 demonstrating approximately 50% inhibition. CTT2, CTT5, CTT7, and CTT11 all demonstrated <15% average inhibition. The apparent lack of antibody competition with the CTT7 construct is consistent with our previous data indicating that this segment is not exposed to antibody binding on the surface of transfected cells (this work and [29]). CTT1 costaining with anti-120, on the other hand, apparently led to an increase in antibody reactivity, as evidenced by negative inhibition. These results indicate that CTT-exposed Env evidently constitutes 20–50% of total Env on the cell surface.

### The CTT is exposed on cells infected with wild-type 89.6 virus

**Figure 5:**
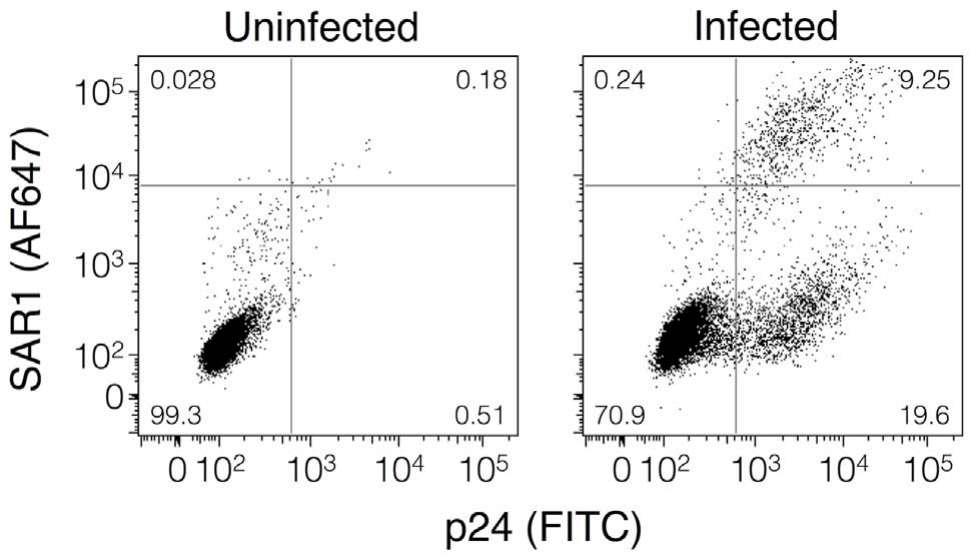
CTT exposure can be detected on virus-infected cells. Unfixed uninfected and HIV-1 89.6-infected CEMx174 cells were surface stained with anti-KE MAb SAR1, and subsequently fixed, permeabilized, and stained with anti-p24 to determine the extent of CTT exposure on virally-infected cells. Dot plots are representative of cells from the live cell population as determined from cellular scatter characteristics.

As the above CTT-exposure experiments were performed on cells expressing codon-optimized Env, there was the possibility that the observed Env expression was a result of Env overexpression. To address this possibility, CEMx174 cells were infected with wild-type 89.6 virus and assayed for the ability of CTT-specific MAb SAR1 to bind to live, intact cells. In cells that stained positive for p24, 32% demonstrated reactivity to anti-CTT monoclonal antibody SAR1 (Figure 5). Uninfected (p24 negative) cells did not demonstrate any apparent exposure of CTT sequences (Figure 5, upper left quadrant). Exposure of the CTT on virally-infected cells indicates that the observed exposure in transfected cells expressing VSV-G-tagged Env was not an artifact of overexpression, and that CTT exposure is apparent during normal viral replication in infected cells. Additionally, CTT-exposed Env was not apparent on the surface of uninfected cells (p24 negative) from the same infected culture, indicating CTT-exposed Env is not likely the result of Env shedding from infected cells.

### CTT exposure is not detected on the surface of VSV-G-tagged pseudovirus particles

**Figure 6:**
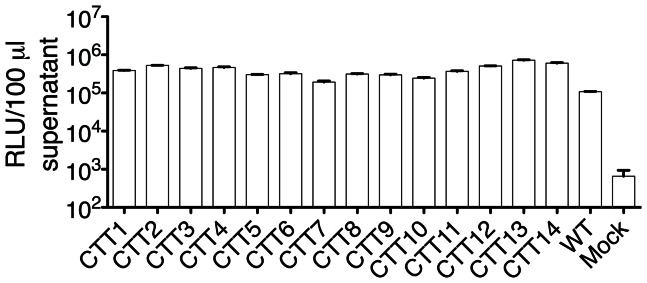
Relative infectivity of pseudoviruses containing VSV-G-tagged Env. Supernatants from HEK293T/17 cells cotransfected with pSG3ΔEnv and the indicated Env construct were used to infect TZM-bl cells to determine infectivity of the resulting pseudovirus particles. Results are presented as relative light units (RLU) per 100 µl of transfection supernatant.

To provide a direct comparison of the VSV-G epitope tag-based topology mapping studies on the cell surface, pseudoviruses were made by cotransfecting the VSV-G-tagged Env with pSG3ΔEnv. This approach has been widely used to generate infectious pseudoviruses for use in neutralization assays [42], [43]. Infectivity of the resulting transfection supernatants was determined using the TZM-bl assay, and results are presented in Figure 6. All VSV-G-tagged Env clones yielded infectious pseudovirus when transfected with pSG3ΔEnv. Luciferase activity from all the clones was >200-fold higher than that observed for the mock transfection and of the same order of magnitude as the wild-type Env. These results suggest that the VSV-G tag substitutions did not evidently interfere with the functional activity of Env, as the resulting pseudovirions were infectious at a similar level to the wild-type Env from which they were derived. As such, the resulting pseudovirus particles were used to map the topology of the CTT by immunoprecipitation using the VSV-G epitope tag as the potentially exposed target.

**Figure 7:**
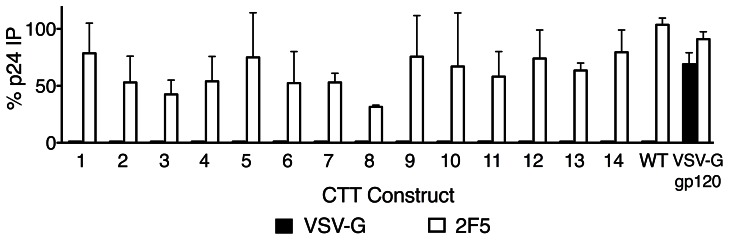
Immunoprecipitation of VSV-G tagged pseudoviral particles. Pseudovirus particles were immunoprecipitated using monoclonal antibodies specific for the VSV-G epitope tag (closed bars) or the gp41 MPER (2F5, open bars). Results are presented as the percent of input p24 that was immunoprecipitated (IP).

Immunoprecipitations were performed as described previously [29]. As a control for antibody accessibility to an exposed membrane-localized epitope, immunoprecipitations with the MPER-directed antibody 2F5 were performed in parallel with the anti-VSV-G pull-downs. As these MAbs are Env-directed, precipitation of intact virions was determined by measurement of the amount of p24 bound to MAb-coated beads relative to the input p24. The rationale is that p24 could only be bound to the beads if the particular MAb of interest bound to an intact virion. Results from the immunoprecipitation assays are presented in Figure 7. The MPER-directed MAb 2F5 was observed to immunoprecipitate intact particles from all VSV-G-tagged pseudoviral clones (32–80% of input p24, depending on the specific construct). In contrast, however, the anti-VSV-G MAb did not pull down p24 from any of the pseudoviral clones. The anti-VSV-G MAb precipitated p24 in pseudoviral particles where the VSV-G epitope tag was inserted into gp120 V3 (VSV-G gp120), demonstrating that the antibody is able to bind and immunoprecipitate pseudovirion particles under these experimental conditions if the epitope is exposed. These results suggest that the CTT sequences are not apparently exposed on the surface of intact virions, in distinct contrast to the observed exposure on the cell surface.

### Dynamic Exposure of CTT Sequences During Viral Infection

**Figure 8:**
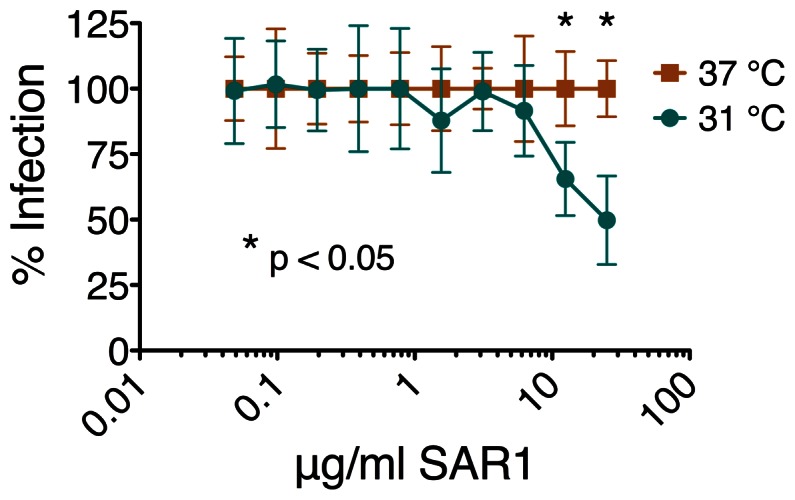
Post-attachment neutralization of HIV-1 89.6 by anti-CTT antibody. Anti-CTT (Kennedy epitope-specific) monoclonal antibody SAR1 was used to determine post-attachment neutralization (PAN) activity at 37°C and 31°C. SAR1 did not exhibit PAN at 37°C, but there was a statistically significant reduction in viral infection when SAR1 was tested for PAN at 31°C. * indicates statistical significance at p<0.05.

The current results demonstrate that a majority of the CTT is exposed to antibody binding in a portion of Env expressed on the cell surface, but is apparently not natively exposed on the virion. To address the potential for CTT sequences to demonstrate transient exposure, post-attachment neutralization (PAN) assays were performed using a monoclonal antibody (SAR1) directed to the Kennedy epitope in the CTT (Figure 8). The assay was performed at both 37°C and 31°C, as prior studies demonstrated PAN activity at temperatures that do not support membrane fusion [27]. Results from this assay demonstrate that SAR1 can mediate PAN when antibody and virus are incubated with cells at 31°C, but not 37°C. These results indicate that CTT sequences that are not natively exposed on the virus become exposed when the virus-cell fusion process is delayed by incubation at lower temperatures.

## Discussion

The current data extend previously published work from our lab on the cell surface exposure of the Kennedy epitope to a study of the topology of the entirety of the CTT sequence. In the current study, we have demonstrated a differing membrane topology for the HIV gp41 CTT on the cell surface compared to that which is present on the surface of infectious virions. On the cell surface, most of the CTT sequence appears to be accessible to antibody binding on the extracellular side of the membrane, while on the virion, no exposure of CTT sequences is detected. These results reiterate the idea that the topology of the CTT of gp41 is more complex than the prevailing model of an exclusively cytoplasmic tail or of a rather limited CTT exposure [28]. While the current data clearly demonstrate the exposure of most of the CTT in Env expressing cells, the data also suggest co-existence with Env proteins containing an intracellular CTT. This concept of mixed populations of cell-surface Env with differing CTT topologies adds an additional level of complexity to studies of the structural and functional roles of the CTT, and it is interesting to consider the biological implications of these two distinct Env species. Following are thoughts on how these data and concepts fit into the current state of CTT functional studies.

The simplest explanation for the discrepancy between the current data and the traditional model is that the CTT-exposed Env is either misfolded or an alternatively folded state. Misfolding of the Env could be due to protein overexpression in transfected cells. However, in general, overexpression of membrane proteins that overwhelm the synthetic machinery tend to lead to the cessation of new protein synthesis concomitant with the induction of the unfolded protein response (UPR) and ER-associated degradation (ERAD) [44]–[49]. If the UPR and ERAD cannot successfully reduce the accumulation of misfolded protein, the cell ultimately undergoes apoptosis [46]. As the current results were determined on intact live cells, the observed CTT exposure is not thought to be an artifact of misfolded protein due to overexpression. In addition, similarly observed exposure of the KE to antibody binding in virally-infected cells (c.f. Figure 5) suggest that CTT exposure is not due simply to the accumulation of misfolded protein as a function of protein overexpression.

A second explanation is that the observed CTT-exposed Env is the result of an alternative folding of the Env with respect to its membrane topology. The idea of multiple folds for membrane proteins is not without precedent (reviewed in [50]). For example, the large envelope glycoprotein of hepatitis B virus is initially inserted in the membrane during biosynthesis with a cytoplasmic N-terminus [51], [52]. Following post-translational maturation, the N-terminus is translocated across the membrane in approximately 50% of the molecules by the insertion of another membrane helix [51], [52]. It is possible that during HIV Env biosynthesis a similar process occurs, resulting in a population of Env with both cytoplasmic and extracellular CTT sequences. Under this assumption, the CTT-exposed Env may be similar to the flavivirus E^rns^ proteins that are attached to the membrane by an in-plane membrane anchor [53], [54]. It is important to note that the current data do not exclude the presence of cell-surface Env with cytoplasmically-localized CTT sequences; the data only demonstrate that CTT-exposed Env exists on the surface of Env-expressing cells. It is possible that both cytoplasmic and extracellularly-localized CTT exists at the same time in the same cell, and that CTT-exposed Env accumulates on the surface of the cell because it cannot be endocytosed due to extracellular localization of the functional endocytic signals [17], leading to enhanced detection.

In addressing the extent of surface-exposed CTT, we found that a substantial portion of the Env on the intact cell surface was CTT-exposed. Under our experimental conditions, prior binding by anti-VSV-G (CTT) antibody reduced anti-HA (gp120) staining by up to 50%. If CTT sequences are exposed on the cell surface during the course of a viral infection, as they appear to be for the Kennedy epitope (Figure 5), the CTT could be an important target for antibody and cytotoxic T-cell responses. The current and previous results have demonstrated that anti-Kennedy epitope MAbs can mediate post-attachment neutralization of viral infection [27], [34]. In addition, the CTT exhibits greater sequence conservation than gp120 [23]. These results, in light of the demonstrated cell-surface CTT exposure, suggest that the in vivo exposure of CTT sequences on the surface of infected cells may provide the immune stimulus necessary to generate an anti-HIV antibody response. It is important to remember that early studies demonstrated the presence of anti-CTT antibodies in patient sera [32], [55]. The exposure of CTT sequences on the surface of infected or transfected cells may also in part explain the exceptionally high immunogenicity observed for this segment of the gp41 protein, especially the KE epitope [32], [55].

In contrast to the exposure of the CTT on the cell surface, we were not able to detect CTT exposure on the virion. Additionally, the fact that we were able to make infectious pseudoviruses with Env proteins containing the VSV-G tag suggests that the Env incorporated into particles is properly functional. Production of infectious pseudoviruses suggests that functional redundancy might be encoded in the CTT, a concept also suggested by previous results demonstrating a need to mutate two endocytic sequences in the CTT to fully abrogate trafficking of Env to late endosomes [17].

Observed topological differences in the CTT between the cell and virion surfaces leads to questions regarding the mechanism by which the observed differences occur. There are two potential explanations: (1) the CTT-exposed Env is incorporated into the budding virion, and undergoes topological rearrangements to localize to the interior of the viral membrane; or (2) the CTT-exposed Env is not incorporated into the budding virion. While (1) is possible, we believe that (2) is the more plausible explanation. However, the true mechanism remains to be determined.

A number of studies have demonstrated a functional interaction of the CTT with proteins localized exclusively in the cellular cytoplasm [9], [10], [13]–[18], [25]. The simplest argument for the exclusion of CTT-exposed from the budding virion is that in this arrangement the CTT cannot interact with known or unknown intracellular partners, and thus does not traffic properly into the viral budding site. However, this simple explanation is sufficient to explain the exclusion of CTT-exposed Env from the virion only to the extent that Env incorporation is an active, regulated process. Evidence from CTT-deleted Env constructs suggests that active Env incorporation is not always necessary. It has been previously demonstrated that viruses encoding a truncated CTT of 17 amino acids past the putative membrane spanning domain retain full infectivity in vitro [56]–[58]. Since CTT-deleted Env cannot be actively incorporated into the budding virion through interactions with intracellular partners such as Gag, it must be incorporated passively [59]. If CTT-deleted Env can be passively incorporated into budding virions, why then is the CTT-exposed Env apparently not?

One hypothesis is that the CTT-exposed Env may be prevented from incorporating into the budding virion due to an interaction with another cell surface protein. In contrast to truncated Env, the CTT-exposed Env contains an additional ∼130 amino acids that contain sequences that have been demonstrated to interact with cellular partners such as calmodulin [60], [61], TAK1, part of the canonical NF-κB pathway [62], and Lumen [63]. As we are just beginning to understand the extent of cell surface protein-protein interactions [64], [65], the presence of a large extracellular sequence as demonstrated for CTT-exposed Env may provide the physical substrate necessary for interactions with as yet undefined cellular partners that prevent segregation into viral budding sites.

Another possible explanation may be based in lipid content differences that have been demonstrated between the T cell membrane and the viral membrane [31], [66]. The HIV membrane contains a higher molar percentage of cholesterol, which has been shown to alter the physical properties of biological membranes in a manner that is dependent on the nature of the membrane phospholipid [67]. As there are differences in the phospholipid content between the T cell membrane and the HIV membrane [31], [66], the increased cholesterol content in the HIV membrane may alter the physical and chemical properties of the membrane in such a way as to disallow the segregation of CTT-exposed Env into the cholesterol-rich budding sites.

In addition to the complications resulting from differential cell surface and virion CTT exposure, the transient exposure of CTT sequences as demonstrated by use of the PAN assay adds a further level of complexity to the topic of CTT topology. Transient exposure of CTT sequences during membrane fusion may provide a mechanistic rationale for the previously demonstrated conservation of arginine relative to lysine in the CTT [23]. In order to become transiently exposed during the virus-cell fusion process, CTT sequences would need to traverse the lipid bilayer from their intravirion localization. Arginine has been demonstrated to facilitate translocation of both soluble small peptides and large proteins through lipid bilayers [68]–[71]. It is possible that the functional consequence of arginine conservation is to facilitate the movement of CTT sequences through the membrane during virus-cell fusion. The functional requirement for CTT translocation remains to be mechanistically defined.

The results presented here introduce a new concept to the topology of the CTT, in particular that the CTT has the potential to exist in two distinct states that can differ between Env-expressing cells and the virion surface. Combined with the apparently transient exposure of CTT sequences during virus-cell fusion, the current studies highlight that the CTT sequence is more complex, both functionally and structurally, than the current intracytoplasmic model suggests, warranting further studies of CTT functions and co-factor interactions.

## Materials and Methods

### Cell and virus constructs

HEK293T/17cells (ATCC) and TZM-bl cells (from the NIH AIDS Reference and Reagent Program) were maintained in DMEM (Life Technologies) supplemented with 10% (v/v) FBS. CEMx174 cells (from the NIH AIDS Reference and Reagent Program) were maintained in RPMI 1640 (Life Technologies) supplemented with 10% (v/v) FBS. Cells were used at passage numbers less than 30. The pSG3ΔEnv used for pseudovirus production was provided by the NIH AIDS Reference and Reagent Program. Infectious HIV-1 89.6 virus was produced by transient transfection of HEK293T/17 cells with the plasmid p89.6 (NIH AIDS Reference and Reagent Program).

### VSV-G epitope tag substitutions

The VSV-G epitope tag (amino acid sequence YTDIEMNRLGK) was cloned serially into the CTT of HIV-1 89.6 Env as previously described [29], and shown schematically in Figure 1. Briefly, the codon-optimized Env gene was cloned into a p2CI vector derived from PCR2.1 by insertion of the CMV promoter and polyA signal sequence from pcDNA3.1(hygro) (Life Technologies) and PCR-amplified IRES-Neomycin resistant sequence from pFB-Neo-LacZ vector (Stratagene). Overlapping PCR was used for the construction of the VSV-G substitution mutants of gp160. The two hybrid primers were constructed containing VSV-G tag sequences at the 5′-ends and HIV gp160 specific sequences at the 3′-ends of both primers used for the substitutions. Final overlapping PCR products were then subcloned into the HIV-1 gp160 expression vector using the appropriate restriction enzymes and the Rapid DNA Ligation Kit (Roche Applied Science). A similar procedure was used to insert the HA tag (amino acid sequence YPYDVPDYA) into gp120 V3 to allow for the determination of total Env surface expression. All VSV-G and HA substitutions were verified by DNA sequencing.

### Cell culture and transfections

HEK293T/17 cells were plated into six-well plates 24 hours prior to transfection. For evaluation of VSV-G epitope exposure on Env-expressing cells, 80% confluent cells were transfected with 2.5 µg of the selected HIV-1 Env mutant DNA using Lipofectamine™ LTX reagent and PLUS™ reagent, as recommended by the manufacturer (Life Technologies). CEMx174 cells were infected with 89.6 virus at an MOI of 0.01 seven days prior to staining.

### Cellular FACS analysis

FACS analysis of Env-expressing cells was carried out as previously described [29]. Briefly, cells were harvested 24 hours post-transfection by treatment with 2 mM EDTA. Cells were resuspended and washed twice with FACS wash buffer (1X PBS with 5% FBS) at 4°C prior to antibody staining. Reference MAbs specific for HA (Roche Diagnostics), VSV-G (Roche Diagnostics), and gp41 (SAR1) were labeled immediately prior to staining using Zenon labeling kits (Life Technologies) following manufacturer instructions. A sample of 10^6^ cells was stained by incubating with 5 µg fluorophore-labeled antibody (HA/VSV-G/gp41-specific) for 30 minutes on ice. Following staining, cells were washed thrice with FACS wash buffer at 4°C. Washed cells were resuspended and stained with 7-amino-actinomycin D (7-AAD). To minimize the potential for both conformational changes as well as reduced antigenicity induced by fixative [72], [73], cells were not fixed prior to analysis. Fluorescently-labeled cells were analyzed on a FACSAria (BD Biosciences). Live intact cells (7-AAD negative) were selected for scatter characteristics, including selection of single-cell populations by doublet-discrimination analysis. PMT settings were adjusted on identically-stained mock transfected cells prior to analysis of Env-transfected cells. Data was collected for ≥5×10^4^ 7-AAD negative cells and analyzed for reactivity with fluorescently-labeled HA, VSV-G, or gp41 antibodies. Passive transfer studies of Env were performed as previously described [36], with staining as described above.

### Production of pseudoviral particles with VSV-G tagged Env

To produce VSV-G tagged pseudovirions, HEK293T/17 cells were plated and transfected as above, with 1.25 µg VSV-G tagged Env DNA and 1.25 µg pSG3ΔEnv. Cellular supernatants were harvested 48 hours post-transfection and clarified at 1,500×g for 10 minutes. The clarified supernatant was pelleted for 1.5 hours at 21,000×g over a 20% glycerol cushion. The pelleted virus was resuspended in 1X PBS and further purified on a 30%/45% sucrose step gradient. The viral band was collected, pelleted over 20% glycerol, and resuspended at a 1000X concentration in 1X PBS.

### Pseudoviral immunoprecipitation and Western blotting

Protein G Dynabeads (Life Technologies) were prepared according to the manufacturer's directions. Briefly, anti-Env or anti-Gag antibodies (4 µg) were incubated with 20 µl protein G Dynabeads in 35 µl citrate-phosphate buffer, pH 5.0, with gentle shaking for 45 minutes at room temperature. Isotype-matched IgG controls were used for each species (murine, human, etc.) from which a MAb was derived. Beads were washed thrice with 0.5 ml citrate-phosphate buffer followed by resuspension in either 26 µl PBS (for intact virus) or 26 µl PBS with 1% Triton X-100 (for lysed virus) and 4 µl pseudovirus. Virus-bead suspensions were incubated at 4°C for one hour with gentle shaking and subsequently washed thrice with 1X PBS. Following the final wash, beads were resuspended in NuPAGE SDS-PAGE buffer, heated at 70°C for 10 minutes, and the supernatant loaded onto 4–12% Bis-Tris NuPAGE gels. Gels were electrophoresed followed by transfer to polyvinylidene fluoride (PVDF) membranes using the Life Technologies iBlot system. Blots were blocked for one hour in 5% blotto (1X PBS with 5% dry milk). After blocking, blots were cut to allow separate staining of gp120 (>60 kDa), gp41 (30–60 kDa), and p24 (<30 kDa). gp120 was stained with rabbit anti-gp120 (Advanced Biotechnologies, Inc.), gp41 stained with Chessie 8, and p24 stained with Ag3.0 for 1.5 hours at room temperature. Blots were washed thrice with 1X PBS and 0.025% Tween 20 (PBS-T), followed by incubation with appropriate secondary antibody (anti-rabbit IgG or anti-mouse IgG conjugated to horseradish peroxidase) for one hour at room temperature. Blots were washed thrice in PBS-T with the gp120 blot receiving an additional wash in 1X PBS with 0.1% Triton X-100. Finally, blots were incubated with PicoWest substrate (Pierce) for one minute and reassembled for visualization on X-ray film.

### Western blot quantitation

Antibody IPs were quantified by densitometry analysis. For each IP, three independent X-ray exposures were scanned and analyzed using ImageJ (NIH). When applicable, p24 bands were selected for each protein, and the integrated area under the densitometry curve was compared to that of the viral input band to yield percent of the input protein in the immunoprecipitate. Percent inputs for each protein for each of the three exposures were averaged to yield the overall percent input immunoprecipitated per experiment. This was repeated for three independent experiments, and the results were averaged to yield the final percent input immunoprecipitated per antibody.
